# Effects of Flavanone Derivatives on Adipocyte Differentiation and Lipid Accumulation in 3T3-L1 Cells

**DOI:** 10.3390/life14111446

**Published:** 2024-11-07

**Authors:** Yasuhito Nobushi, Taira Wada, Motofumi Miura, Rikuto Onoda, Ryuta Ishiwata, Naoki Oikawa, Karin Shigematsu, Toshinori Nakakita, Masaharu Toriyama, Shigeki Shimba, Yukinaga Kishikawa

**Affiliations:** 1 Laboratory of Clinical Pharmacy, School of Pharmacy, Nihon University, 7-7-1, Narashinodai, Funabashi 274-8555, Chiba, Japan; phri18057@g.nihon-u.ac.jp (R.O.); phry18021@g.nihon-u.ac.jp (R.I.); kishikawa.yukinaga@nihon-u.ac.jp (Y.K.); 2 Laboratory of Health Science, School of Pharmacy, Nihon University, 7-7-1, Narashinodai, Funabashi 274-8555, Chiba, Japan; wada.taira@nihon-u.ac.jp (T.W.); shimba.shigeki@nihon-u.ac.jp (S.S.); 3 Laboratory of Molecular Chemistry, School of Pharmacy, Nihon University, 7-7-1, Narashinodai, Funabashi 274-8555, Chiba, Japan; miura.motofumi@nihon-u.ac.jp (M.M.); shigematsu.karin@nihon-u.ac.jp (K.S.); toriyama.masaharu06@nihon-u.ac.jp (M.T.); 4 Laboratory of Medicinal Chemistry, School of Pharmacy, Nihon University, 7-7-1, Narashinodai, Funabashi 274-8555, Chiba, Japan; oikawa.naoki@nihon-u.ac.jp; 5 Medicine Analysis Research Laboratory, Yokohama University of Pharmacy, 601, Matano-cho, Totsuka-ku, Yokohama 245-0066, Kanagawa, Japan; toshinori.nakakita@hamayaku.ac.jp

**Keywords:** flavanone derivatives, adipocyte differentiation, obesity, 3T3-L1 cells

## Abstract

Flavanones, a class of flavonoids, are abundant in fruits, vegetables, and herbs. They are known to have several biological activities, such as anti-inflammatory and anti-cancer activities, but their effects on obesity remain unclear. Obesity is closely associated with adipocyte differentiation and lipid accumulation in adipose tissue. Therefore, in this study, we examined the effects of flavanone derivatives on adipocyte differentiation and lipid accumulation by using 3T3-L1 cells. Among the 15 flavanone derivatives studied, 4′-phenylflavanone (4PF), with a biphenyl structure, significantly inhibited adipocyte differentiation-related lipid accumulation in 3T3-L1 cells; this inhibition of lipid accumulation was dose-dependent. Gene expression analysis showed that 4PF suppressed the expression of adipogenic marker genes. Although the induction of *peroxisome proliferator activator γ2* (*Pparγ2*), a master regulator of adipocyte differentiation, and its target genes during adipocyte differentiation was attenuated in 4PF-treated cells, 4PF did not directly regulate *Pparγ2* gene expression and its activation. In contrast, 4PF suppressed mitotic clonal expansion (MCE), which is associated with changes in the expression of proliferation-related genes at the early stages of adipocyte differentiation. Taken together, these results suggest that 4PF inhibits lipid accumulation because it suppresses MCE during adipocyte differentiation. Thus, our findings may help in the development of anti-obesity drugs.

## 1. Introduction

Obesity, a global public health concern for decades, is associated with the development of metabolic diseases such as type 2 diabetes, dyslipidemias, hypertension, and atherosclerosis [1,2,3]. Obesity is the result of an imbalance between energy intake and energy expenditure, leading to metabolic dysfunction and excess fat accumulation in adipose tissue [4]. Indeed, excess fat accumulation occurs due to an increase in the number of adipocytes (hyperplasia) and the size of adipocytes (hypertrophy). Hyperplasia is related to the generation of new adipocytes from precursor cells, called adipogenesis. Adipogenesis is the process by which preadipocytes, which are precursor cells, differentiate into adipocytes. Therefore, the inhibition of adipocyte differentiation could constitute an advantageous strategy for developing anti-obesity agents [5].

3T3-L1 cells are a convenient in vitro model system to study the mechanism underlying adipocyte differentiation and adipocyte function, and to screen novel anti-obesity agents [6,7]. Natural products and their derivatives possess diverse biological activities; therefore, they have been investigated to identify beneficial anti-obesity compounds. We have previously shown that natural compound pentagalloyl glucose and non-natural compounds including burchellin derivatives and indirubin-3′-oxime derivatives inhibit lipid accumulation in 3T3-L1 cells [8,9,10].

Flavanones are a group of flavonoids with a C6-C3-C6 skeleton. Flavanones were minor flavonoids a few years ago. However, more than 350 flavanone aglycones and 100 flavanone glycosides have since been identified, and flavanones are now recognized as major flavonoids [11]. Moreover, flavanones are widely distributed as secondary metabolites in fruits and are present in several herbal medicines [12]. Numerous studies have suggested that natural flavanones possess multiple biological activities such as anti-oxidant, anti-inflammatory, anti-cancer, anti-bacterial, and anti-virus activities [13,14,15,16,17]. Naringin and hesperidin are two flavanone glycosides that have been reported to possess anti-diabetic, lipid-lowering, and anti-obesity activities [18,19].

A recent study showed that naringin and its aglycone naringenin inhibit lipid accumulation and adipocyte differentiation [20]. In contrast, sakuranetin (natural flavanone) and flavanone (non-natural flavanone) have been reported to promote adipocyte differentiation and lipid accumulation [21,22]. Moreover, we also reported that three flavanones, cryptostrobin, pinocembrin, and 5,7-dihydroxy-6,8-dimethylflavanone, isolated from the dried branches and leaves of murta (*Myrceugenia euosma*), had no effect on lipid accumulation [23]. Thus, the effect of flavanones on adipocyte differentiation remains controversial.

In this study, to better understand the biological activity of flavanones during adipocyte differentiation, we synthesized 15 flavanone derivatives, including those with an electron-donating group (EDG) or an electron-withdrawing group (EWG), to obtain information on the substituents and electron density of the aromatic ring of the flavanone skeleton, and then investigated those effects on lipid accumulation and adipocyte differentiation in 3T3-L1 cells.

## 2. Materials and Methods

### 2.1. Flavanone Derivatives

**Figure 1:**
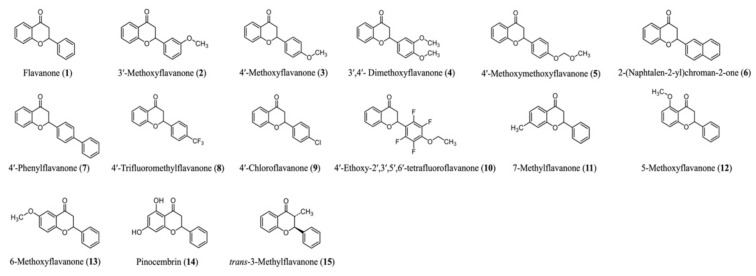
Structures of the flavanone derivatives (**1**–**15**) used in this study.

The structures of the natural flavanone (**14**) and non-natural flavanone derivatives (**1**–**13**, **15**), which were prepared as previously reported, are shown in Figure 1 [24]. In brief, these flavanones were readily synthesized using a catalytic amount of cesium fluoride aqueous solution from corresponding chalcones, which were products of benzaldehyde derivatives and 2′-hydroxyacetophenone derivatives via aldol condensation.

### 2.2. Cell Culture and Adipocyte Differentiation

Mouse 3T3-L1 cells and human hepatoblastoma HepG2 cells were obtained from the American Type Culture Collection (Manassas, VA, USA) and the European Collection of Cell Culture, respectively. 3T3-L1 and HepG2 cells were maintained in Dulbecco’s Modified Eagle’s Medium (DMEM; Nissui, Tokyo, Japan) supplemented with 10% calf serum (CS, Thermo Fisher Scientific, Waltham, MA, USA) and 10% fetal bovine serum [FBS; Cosmo Bio, Tokyo, Japan] at 37 °C in 5% CO_2_, respectively.

To induce adipocyte differentiation, confluent 3T3-L1 cells were cultured for 48 h in differentiation medium (DMEM/Ham’s F12 [Nissui, Tokyo, Japan], 10% FBS, 1.6 μM insulin, 0.0005% transferrin, 180 μM adenine, and 20 pM triiodothyronine) with 500 μM isobutyl-3-methylxanthine (IBMX) and 0.25 μM dexamethasone (DEX). The differentiation medium was changed every 2 days. Flavanone derivatives were dissolved in dimethyl sulfoxide (DMSO) and were diluted in differentiation medium to a final concentration of 0.1% on day 0. DMSO-treated cells were used as Control.

### 2.3. Cell Viability Measurement

Cell viability assays were performed using the 3-(4,5-dimethylthiazol-2-yl)-2, 5-diphenyltetrazolium bromide (MTT) assay. 3T3-L1 cells were seeded at a density of 1 × 10^4^ cells/well in 96-well plates (IWAKI, Asahi Glass Co., Ltd., Tokyo, Japan) and cultured in DMEM supplemented with 10% CS for 2 days. The cells were then treated with flavanone derivatives or DMSO (Control) for 48 h. The MTT Cell Count Kit (Nacalai Tesque, Inc., Kyoto, Japan) was used according to the manufacturer’s instructions. Absorbance was measured at 570 nm using a model 680XR microplate reader (Bio-Rad Laboratories, Hercules, CA, USA).

### 2.4. Oil Red O Staining

Oil red O staining was performed as previously described [9]. Briefly, differentiated 3T3-L1 cells were washed once with phosphate-buffered saline (PBS) and fixed with 4% formaldehyde solution at room temperature for 24 h. The fixed cells were then rinsed once with water and stained with oil red O solution from a Lipid Assay Kit (Cosmo Bio, Tokyo, Japan) at room temperature for 15 min. The stained cells were washed three times and dehydrated for 10 min. Images were obtained using an Olympus IX71 microscope (Olympus, Tokyo, Japan).

For the quantification of lipid droplets, oil red O-stained cells were eluted with solubilization solution and quantified by measuring the absorbance at 540 nm with a FLUOstar Omega system (BMG LABTECH, Ortenberg, Germany).

### 2.5. Quantitative RT-PCR (qRT-PCR)

Total RNA was isolated from 3T3-L1 cells by using RNAiso Plus (Takara, Shiga, Japan), according to the manufacturer’s instructions. cDNA was reverse-transcribed from total RNA (1.0 μg) by using ReverTra Ace RT (Toyobo, Osaka, Japan) and an oligo dT primer. PCR was performed with the Mx3000 quantitative RT-PCR system (Agilent Technologies, Santa Clara, CA, USA), using SYBR Green PCR reagents (Promega, Madison, WI, USA), primers (0.2 μM), and prepared cDNA. The initial thermal conditions were 95 °C for 30 s, followed by 40 cycles of initial denaturation at 95 °C for 30 s each, annealing at 58 °C for 30 s, and elongation at 72 °C for 30 s. The relative mRNA expression levels of the target genes were determined using the 2^−ΔΔCt^ method, with 36B4 as the reference gene [25]. The primer sequences used for qRT-PCR are listed in Table 1.

### 2.6. Plasmid Constructs, Cell Transfection, and Mammalian Two-Hybrid System

For the mammalian two-hybrid assay, the pG5 UAS Luc reporter vector (Promega, Madison, WI, USA) containing five copies of the GAL4 upstream activating sequence (UAS) and the pBind vector containing the yeast GAL4 DNA binding domain were obtained from Promega. The region of human peroxisome proliferator activated receptor γ (PPARγ) ligand-binding-domain (LBD, amino acids 157–475, accession number: L40904) was amplified by PCR using human PPARγ expression plasmid as the PCR template and the following oligonucleotides: PPARγ LBD forward-5′-AATAAATGTCAGTACTGTCG-3′ and reverse-5′-CTAGTACAAGTCCTTGTAGATCTCCTGCAG-3′ [26]. The PCR-amplified fragment digested with the SalI and KpnI restriction enzymes was cloned into the pBind vector (GAL4 PPARγ LBD). tk-PPRE Luc is a luciferase reporter containing the herpes virus thymidine kinase promoter downstream of three copies of peroxisome proliferator response elements (PPREs) from the acyl-CoA oxidase gene. 3T3-L1 cells in 48-well plates were transfected with plasmid pG5 UAS Luc, GAL4 PPARγ LBD, and pGL4.75 vectors for the mammalian two-hybrid assay and tk-PPRE Luc, mouse PPARγ2 expression, pRC/CMV2 empty, and pGL4.75 vectors for the reporter assay using Lipofectamine 2000 (Thermo Fisher Scientific, Waltham, MA, USA), according to the manufacturer’s instructions [27]. After 6 h of incubation, the transfection medium was replaced with fresh DMEM medium. On the next day, the medium was replaced with fresh medium containing PPARγ ligand, pioglitazone (FUJIFILM Wako Pure Chemical Co., Ltd., Osaka, Japan), antagonist GW9662 (FUJIFILM Wako Pure Chemical Co., Ltd., Osaka, Japan), and 4PF (**7**) for 18 h. Cell lysates were extracted and assayed using a dual luciferase reporter assay system (Promega, Madison, WI, USA). The pGL4.75 vector was used as a normalization control to correct for variable transfection efficiencies. All transfections were performed in triplicate.

### 2.7. Cell Proliferation Assay

Adipocyte differentiation was induced for 3T3-L1 cells seeded in a 24-well plate (Iwaki) as described under cell culture and adipocyte differentiation. HepG2 cells were seeded at a density of 3 × 10^4^ cells/well in a 24-well plate (Iwaki) before the assay. The cells were washed in PBS and harvested by incubating at 37 °C for 5 min with trypsin. Viable cells were counted using the trypan blue exclusion method with a hemocytometer.

### 2.8. Statistical Analysis

Statistical analysis was performed using Minitab v.18 (Minitab, Inc., State College, PA, USA). The results were expressed in terms of mean ± standard deviation (SD) values of three experiments. Student’s *t*-tests were performed for comparisons between two groups. One-way analysis of variance (ANOVA) was performed for comparison between three or more treatments with Tukey’s post hoc test. Two-way ANOVA was performed on the treatment and another treatment using Tukey’s post hoc test. Statistically significance was set at *p* < 0.05.

## 3. Results

### 3.1. Effects of Flavanone Derivatives on Lipid Accumulation in 3T3-L1 Cells

**Figure 2:**
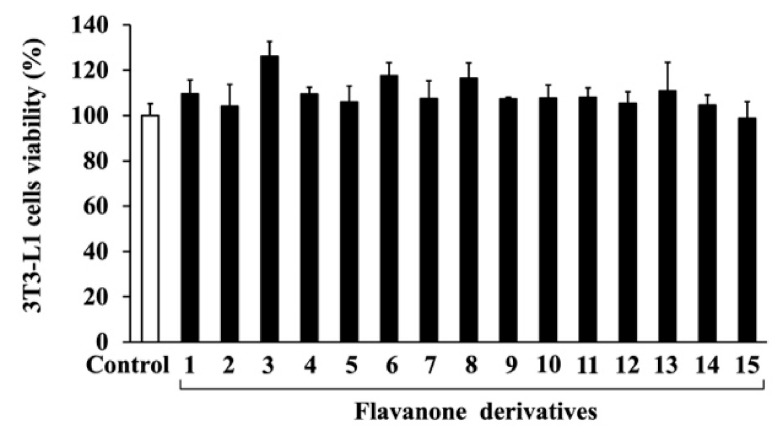
Effects of flavanone derivatives on 3T3-L1 cell viability. 3T3-L1 preadipocytes were incubated with flavanone derivatives (**1**–**15**) (50 μM) or DMSO (Control) for 2 days, and cell viability was determined using the MTT assay. The value of the Control cells was set at 100 (%).

**Figure 3:**
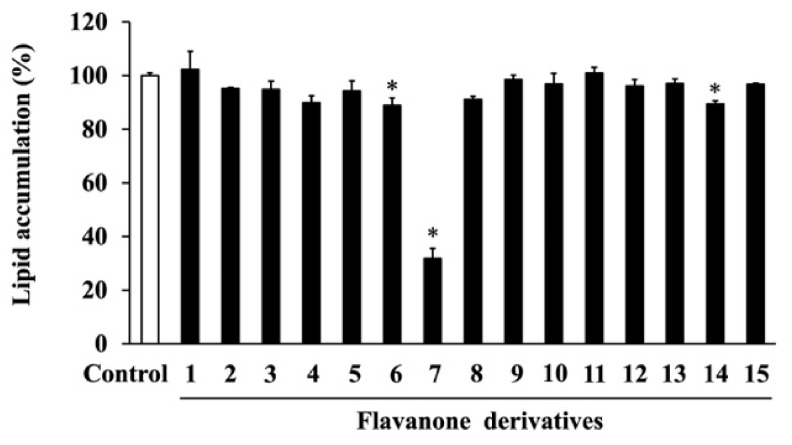
Cells were treated with each of the flavanone derivatives (**1**–**15**) (50 μM) or DMSO (Control) on day 0, 2, and 4 during adipocyte differentiation. On day 6, intracellular lipids stained with oil red O dye were eluted, and their levels were quantified by measuring absorbance at 540 nm. The value of the Control cells was set at 100 (%). Data were represented as the means ± SD values of three independent experiments and were compared using one-way ANOVA with Turkey’s post hoc test. * *p * <  0.05, relative to Control cells.

To investigate the cytotoxicity of the 15 synthesized flavanone derivatives (Figure 1), 3T3-L1 preadipocytes were treated with flavanone derivatives for 2 days, and cell viability was measured using the MTT assay (Figure 2). The flavanone derivatives were not cytotoxic at concentrations up to 50 μM; therefore, they were used at 50 μM in subsequent experiments. To evaluate the effect of flavanone derivatives on lipid accumulation in 3T3-L1 cells during adipocyte differentiation, intracellular lipid content was measured using oil red O staining. Cells treated with 2-naphthylflavanone (**6**), 4′-phenylflavanone (4PF) (**7**), or pinocembrin (**14**) showed significantly less oil red O staining than Control cells (Figure 3). 4PF (**7**) was the most effective inhibitor of lipid accumulation in 3T3-L1 cells; the other flavanone derivatives did not exert an inhibitory effect.

**Figure 4:**
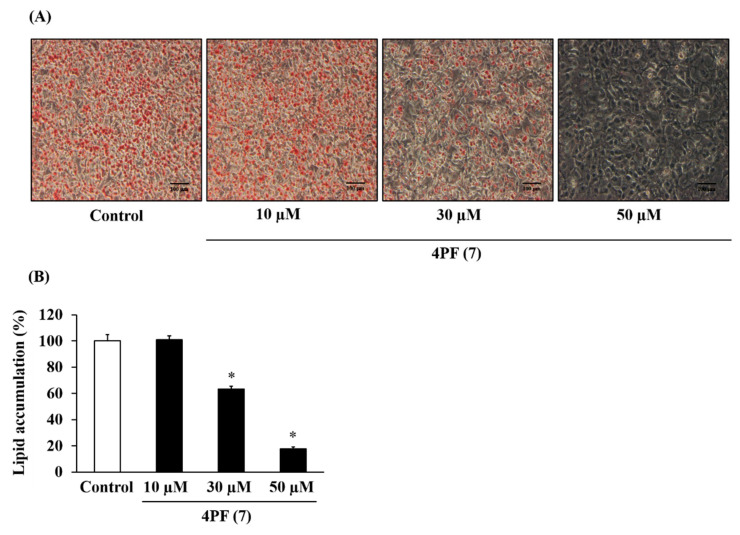
Dose-dependent effect of 4PF (**7**) on lipid accumulation in 3T3-L1 cells. 3T3-L1 cells were treated with 4PF (**7**) or DMSO (Control) at the indicated doses on day 0, 2, and 4 during adipocyte differentiation. (**A**) The cells were stained with oil red O after 6 days of differentiation and were microscopically observed at 100× magnification. (**B**) Intracellular lipids stained with oil red O dye were eluted, and their levels were quantified by measuring absorbance at 540 nm. The value of the Control cells was set to 100 (%). Data were represented as the means ± SD values of three independent experiments and were compared using one-way ANOVA with Turkey’s post hoc test. * *p * <  0.05, relative to Control cells.

Furthermore, this decrease in lipid accumulation due to 4PF (**7**) treatment was dose-dependent (Figure 4). Berberine (BBR), a major activator of Coptidis Rhizoma, is a known inhibitor of lipid accumulation. As shown in Figure S1A,B, BBR treatment at 4 µM also inhibited lipid accumulation.

### 3.2. Effect of 4PF (**7**) on Gene Expression During Adipocyte Differentiation

**Figure 5:**
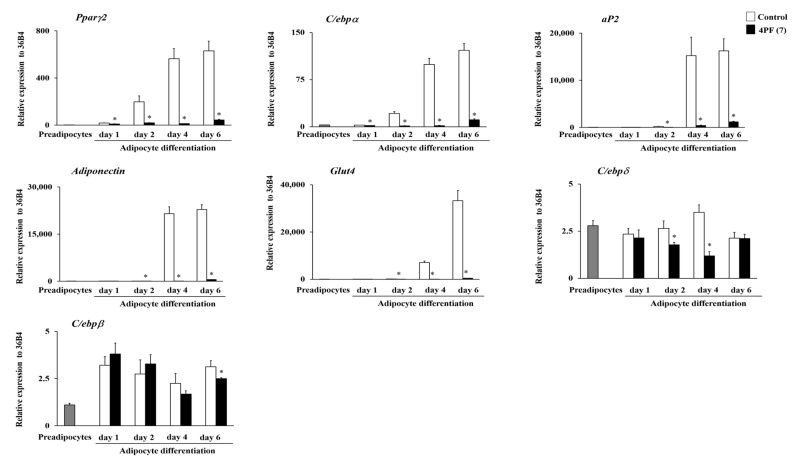
mRNA expression levels of adipocyte differentiation-related genes in 4PF (**7**)-treated 3T3-L1 cells. 3T3-L1 cells were treated with 4PF (**7**) (50 μM) or DMSO (Control) on day 0, 2, and 4 during adipocyte differentiation. Total RNA was extracted at the indicated time points after differentiation, and the expression of adipocyte differentiation-related genes was determined by qRT-PCR. Data were compared using an unpaired Student’s *t*-test. * *p* < 0.05.

Adipocyte differentiation contributes lipid accumulation in adipocytes. To better understand the mechanism underlying the inhibition of lipid accumulation by 4PF (**7**), we examined the expression levels of adipocyte differentiation-related genes. Consistent with lipid accumulation findings, cells treated with 4PF (**7**) had a significantly lower expression of adipocyte marker genes, such as *peroxisome proliferator-activated receptor γ2* (*Pparγ2*), *adipocyte protein 2* (*aP2*), and *CCAAT/enhancer-binding protein α* (*C/ebpα*), than the Control cells (Figure 5). BBR treatment at 4 μM also inhibited adipocyte marker gene expression on day 6 after differentiation (Figure S1). Adipocytes control energy metabolism through adipocytokine secretion and glucose uptake [28,29]. 4PF (**7**) treatment suppressed the induction of *Adiponectin* (adipocytokine) and *glucose transporter 4* (*Glut4*; insulin-dependent glucose transporter) gene expression during adipocyte differentiation. C/EBPβ and C/EBPδ play important roles at the early stages of adipocyte differentiation [30]. *C/ebpδ* gene expression was significantly decreased in 4PF (**7**)-treated 3T3-L1 cells on day 2 after differentiation induction, whereas the *C/ebpβ* expression levels were consistent between Control and 4PF (**7**)-treated cells at the early stages of adipocyte differentiation. Consequently, these results indicate that 4PF (**7**) has inhibitory effect on the adipocyte differentiation in 3T3-L1 cells.

### 3.3. Effect of 4PF (**7**) on the Ligand-Dependent Activation and Gene Expression of PPARγ

**Figure 6:**
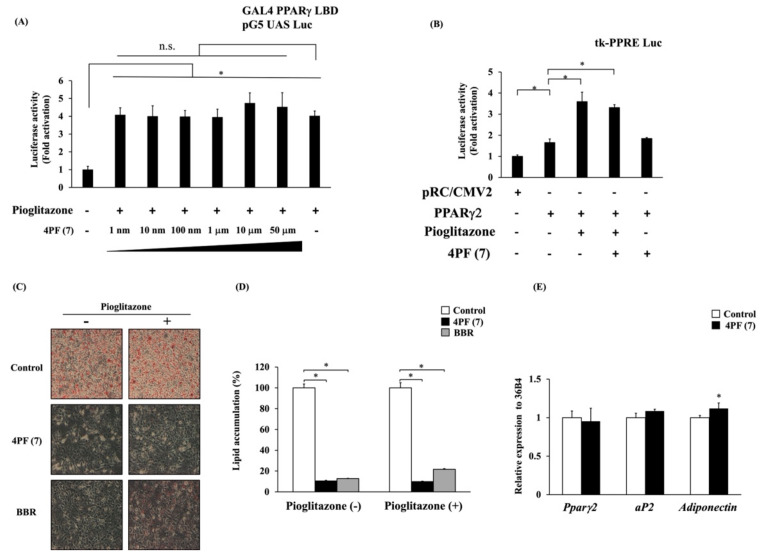
Effect of 4PF (**7**) on ligand-dependent activation and adipocyte differentiation-related gene expression. (**A**,**B**) Luciferase activity in 3T3-L1 preadipocytes transfected with the reporter gene containing pG5 UAS Luc and GAL4 PPARγ LBD vectors (**A**) or tk-PPRE Luc, pRC/CMV2 empty, or PPARγ2 expression vectors (**B**). Transfected cells were treated with indicated concentration of 4PF (**7**), DMSO, and/or pioglitazone 1 μM for 18 h before the luciferase assay. The values of DMSO-treated cells in the absence of 4PF (**7**) and pioglitazone (**A**) and transfected with pRC/CMV2 empty vector (**B**) were normalized to 1. Data were represented as the means ± SD values of three (**A**) and four (**B**) independent experiments and were compared using one-way ANOVA with Turkey’s post hoc test. * *p * <  0.05. n.s: not significant. (**C**) 3T3-L1 cells were treated with differentiation medium containing the indicated reagents, were fixed and stained with oil red O after 6 days of differentiation, and were microscopically observed at 100× magnification. (**D**) Intracellular lipids stained with oil red O dye were eluted, and their levels were quantified by measuring absorbance at 540 nm. The value of the Control cells in the absence of pioglitazone was set to 100 (%). Data were represented as the means ± SD values of three independent experiments and were compared using two-way ANOVA with Turkey’s post hoc test. (**E**) Mature 3T3-L1 adipocytes were treated with 4PF (**7**) (50 µM) or DMSO (Control) for 24 h. Total RNA was extracted and the expression of adipocyte differentiation-related genes was determined by qRT-PCR. The value of Control cells was normalized to 1. Data were compared using an unpaired Student’s *t*-test. * *p* < 0.05.

The result described above indicated that 4PF (**7**) treatment suppressed the expression of the *Pparγ2* gene during adipocyte differentiation. PPARγ2 is a master regulator of adipocyte differentiation and transactivates adipocyte differentiation-related genes. PPARγ2 is a member of the nuclear hormone receptor superfamily of ligand-activated transcription factors. The activation of PPARγ with ligands such as pioglitazone is known to promote adipocyte differentiation in vitro. Then, to investigate the possibility of 4PF (**7**) binding to the ligand binding domain (LBD) of PPARγ as an antagonist, we examined the potential binding of 4PF (**7**) to PPARγ (LBD) by a mammalian two-hybrid system. Pioglitazone-induced GAL4 PPARγ LBD-dependent luciferase activity was not suppressed by 4PF (**7**) at any concentration (Figure 6A). Pioglitazone-induced GAL4 PPARγ LBD-dependent luciferase activity was observed and its induction was suppressed by GW9662, a synthetic antagonist of PPARγ, but not by BBR (Figure S2A). In addition, 4PF (**7**) treatment also had no effect on GAL4 PPARγ LBD-dependent luciferase activity (Figure S2B). 4PF (**7**) had no effect on the PPARγ binding domain, indicating that 4PF (**7**) is not a PPARγ modulator such as an agonist, partial agonist, and antagonist. To further investigate whether 4PF (**7**) affects the transcriptional activity of PPARγ by binding the domain of PPARγ LBD, we assessed the transcriptional activation of peroxisome proliferator response element (PPRE)-driven reporter activity by the overexpression of full-length PPARγ. As shown in Figure 6B, 4PF (**7**) had no effect on the reporter activity by PPARγ with or without pioglitazone.

Endogenous PPARγ ligand production is essential for the adipocyte differentiation process in 3T3-L1 cells, and the suppression of adipocyte differentiation by its inhibition can be restored by the activation of exogenous ligands [31]. Therefore, to examine the effect of 4PF (**7**) on the endogenous PPARγ ligand production, we investigated whether the activation of PPARγ by pioglitazone can restore the differentiation potential of the 4PF (**7**)-treated cells. Pioglitazone could not restore the differentiation potential of the 4PF (**7**)-treated cells nor that of the BBR-treated cells (Figure 6C,D).

It is unclear whether the effect of 4PF (**7**) on adipocyte differentiation-related gene expression is a transcriptional regulation or a result of the suppressed adipocyte differentiation. Therefore, we examined the effect of 4PF (**7**) on adipocyte differentiation-related gene expression in mature 3T3-L1 adipocytes after differentiation. There was no difference in the expression of *Pparγ2*, *aP2*, or *Adiponectin* genes between Control and 4PF (**7**)-treated mature adipocytes (Figure 6E). Consistent with other reports, treatment with GW9662 in 3T3-L1 adipocytes also showed a trend towards a non-significant decrease in the expression of the PPARγ2 gene, but not the adiponectin genes (Figure S2C [32,33]). These results revealed that 4PF (**7**) did not directly regulate *Pparγ* gene expression nor its activation.

### 3.4. Effect of 4PF (**7**) on Mitotic Clonal Expansion (MCE) During Adipocyte Differentiation

**Figure 7:**
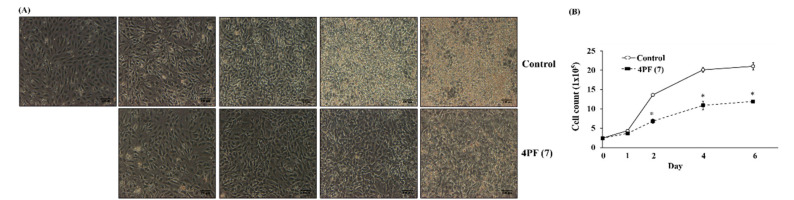

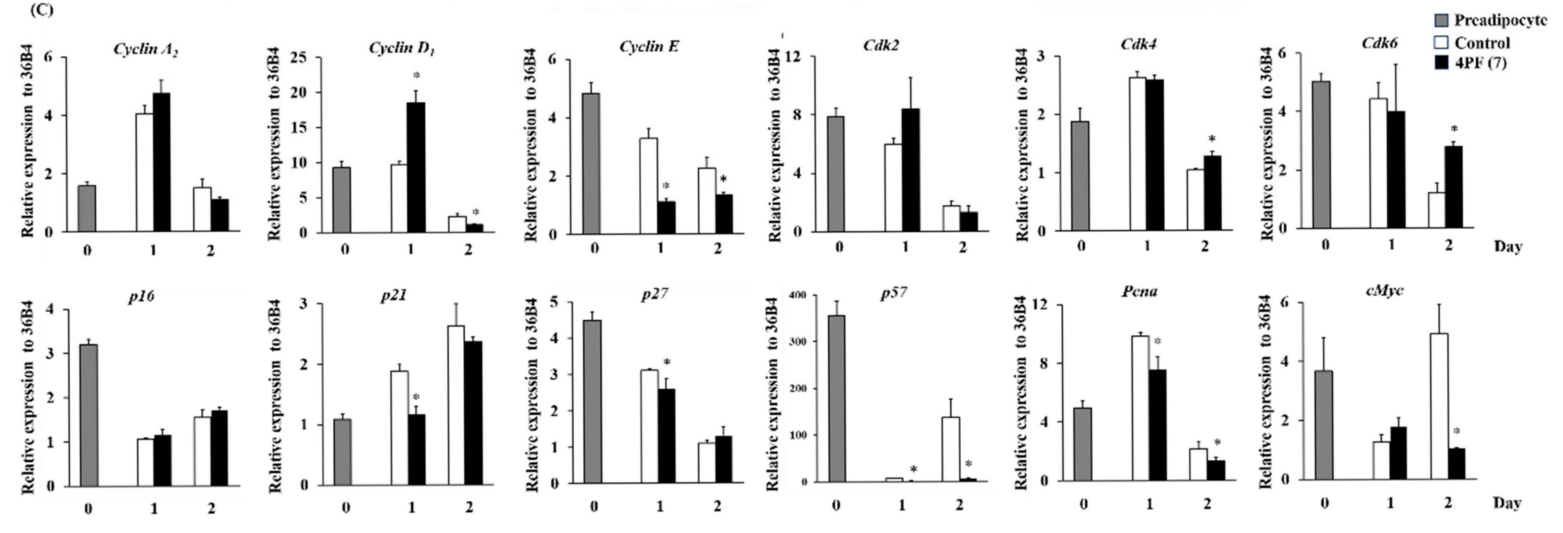
Effect of 4PF (**7**) on mitotic clonal expansion during 3T3-L1 cell differentiation. 3T3-L1 cells were treated with 4PF (**7**) (50 μM) or DMSO (Control) on days 0, 2, and 4 during adipocyte differentiation. (**A**) Changes in cell morphological features were microscopically observed at 100× magnification. (**B**) Cell counts were determined at the indicated time points during adipocyte differentiation. (**C**) The expression of cell cycle-related genes was determined by qRT-PCR. Data were compared using an unpaired Student’s *t*-test. * *p* < 0.05.

MCE is an essential event at the early stages of adipocyte differentiation in 3T3-L1 cells [34]. 4PF (**7**) treatment resulted in changes in cell morphological features and cell number; it suppressed cell growth associated with MCE on day 2 and subsequent changes in cell morphological features (Figure 7A,B). To examine the specific effect of 4PF (**7**) on cell proliferation in 3T3-L1 cells, we performed the cell proliferation assay in human hepatoblastoma HepG2 cells. The results show that 4PF (**7**) suppressed cell proliferation in HepG2 cells (Figure S3). Cell cycle progression is positively controlled by cyclin/cyclin-dependent kinase (CDK) complexes and negatively regulated by its complex inhibitors such as p16, p21, p27, and p57 To further investigate the inhibitory mechanism of 4PF (**7**) on MCE, we examined the gene expression of cell cycle regulators and related factors. The gene expression analysis of the factors associated with cell cycle progression during MCE showed that the expression levels of *Cyclin E*, *p21*, and *p57* in 4PF (**7**)-treated cells were lower than those in Control cells (Figure 7C). In contrast, the expression of *Cdk4* and *Cdk6* genes in 4PF (**7**)-treated cells was increased on day 2 (Figure 7C). *Cyclin D_1_* gene expression was largely altered in 4PF (**7**)-treated cells compared to differentiation in Control cells. Among other cell proliferation-related genes, the expression of *Pcna,* and *cMyc* genes in 4PF (**7**)-treated cells was significantly lower than those in Control cells (Figure 7C). These results suggest that the altered gene expression of cell proliferation by 4PF (**7**) may generate the suppression of MCE and subsequent adipocyte differentiation.

## 4. Discussion

Observational and cohort studies have reported a negative association between flavanone intake and the risk of metabolic diseases such as obesity and type II diabetes as well as flavonoids [35,36]. Obesity is closely associated with the development of metabolic diseases, resulting from adipocyte differentiation and hypertrophy. The anti-obesity effects of whole flavonoids have been extensively studied, while those of flavanones, a subgroup of flavonoids, have not been well investigated. We found that 4PF (**7**) with biphenyl structure significantly reduced lipid accumulation among the natural flavanone (**14**) and non-natural flavanone derivatives (**1**–**13**, **15**), synthesized by the previously established method (Figure 3). 4PF (**7**) inhibited adipocyte differentiation (Figure 5). Furthermore, 4PF (**7**) mechanistically suppressed the proliferation of MCE and the subsequent adipocyte differentiation (Figure 7).

Recently, numerous studies have been conducted to identify anti-obesity components including flavonoids. In this study, 4PF (**7**) inhibited lipid accumulation and adipocyte differentiation as well as BBR (Figure 3, Figure 4, Figure 5 and Figure 6 and Figure S1). Although the concentrations of 4PF (**7**) and BBR were 50 μM and 4 μM, respectively, the concentration of 4PF (**7**) at 50 μM, where the maximum inhibition of lipid accumulation was observed in the current work, was comparable to most of the concentrations used for the inhibitory effect on lipid accumulation and adipocyte differentiation by other flavonoids such as flavanol (nobiletin (100 μM) and fisetin (50 μM)), and flavone (apigenin 50–70 μM) [37,38,39]. Recently, nobiletin and apigenin which are used at high or similar concentrations to inhibit adipocyte differentiation in 3T3-L1 cells, prevented high-fat diet-induced weight gain in in vivo experiments, suggesting the potential of 4PF (**7**) as an anti-obesity compound [40,41]. 4PF (**7**) is a synthetic compound, not a natural one, so safety and other issues need to be considered, but it may be possible to create a more potent compound that has the same effect at lower concentrations through derivatives and other means. In addition, the effect of 4PF (**7**) as an anti-obesity agent should be elucidated in vivo.

Zhang et al. reported that BBR inhibits adipocyte differentiation by suppressing C/EBPβ expression [42]. C/EBPβ expression was not different between Control cells and 4PF (**7**)-treated cells (Figure 5). Thus, it is likely that the mechanism underlying the inhibition of adipocyte differentiation by 4PF (**7**) is different from that of BBR.

PPARγ2 is known as a master regulator of adipocyte differentiation and function [43]. PPARγ2 and C/EBPα regulate each other through positive feedback loops and transactivate downstream target genes such as *aP2*, *Adiponectin*, and *Glut4* [44,45]. 4PF (**7**) treatment suppressed the expression of *Pparγ2* and *C/ebpα* genes at the early stage of adipocyte differentiation and the expression of its target genes, including *aP2*, *Adiponectin*, and *Glut4* at the terminal stage of adipocyte differentiation (Figure 5). On the other hand, 4PF (**7**) treatment did not directly affect the expression of adipocyte differentiation-related genes in mature 3T3-L1 adipocytes (Figure 6), suggesting that the decreased expression of these genes in 4PF (**7**)-treated 3T3-L1 cells during adipocyte differentiation is an indirect result of the decrease in the adipocyte differentiation. Furthermore, some flavonoids, including flavanol, flavone, isoflavone, and flavanone, have been shown to have PPARγ agonist and antagonist activities [22,46,47,48]. As shown in Figure 6B–D, 4PF (**7**) did not show a potential of binding to PPARγ. Although the production of endogenous PPARγ ligand at the early event of differentiation is responsible for the process of adipogenesis [31], exogenous pioglitazone could not restore the potential of adipocyte differentiation in 3T3-L1 treated with 4PF (**7**). This implies that 4PF (**7**) has no effect on the production of endogenous PPARγ ligand (Figure 6). Taken together, these results suggest that 4PF (**7**) regulates the downstream of PPARγ signaling activity not in mature adipocytes but during adipocyte differentiation.

C/EBPβ and C/EBPδ are induced at the early stages of adipocyte differentiation and trigger the induction of PPARγ2 and C/EBPα expression and MCE [30,49,50]. *C/ebpδ* gene expression in the 4PF (**7**)-treated cells was significantly lower than that in Control cells. Hishida et al. reported that the knockdown of the *C/ebpδ* gene resulted in the suppression of *Pparγ2* and *C/ebpα* gene expression and of MCE [30]. MCE is responsible for the induction of the PPARγ2 and C/EBPα genes and the promotion of cell morphological features such as lipid accumulation [49,50]. As shown in Figure 7, 4PF (**7**) suppressed cell proliferation related to adipocyte differentiation and cell morphological changes on day 2. On the other hand, cell proliferation in HepG2 cells was almost completely inhibited on day 2, indicating that more than 24 h exposure to 4PF (**7**) was required to inhibit cell growth. During MCE, the disruption of cell cycle-related gene expression was observed in 4PF (**7**)-treated cells. Consistent with cell proliferation, the expression of *Cyclin E*, *Cyclin D_1_*, and *cMyc* genes was significantly decreased in 4PF (**7**)-treated cells on day 2 (Figure 7). These three genes are targets of E2F which play an important role in G1/S transition and DNA synthesis as well as adipocyte differentiation [51]. cMyc is required for cell proliferation and its downregulation is essential for adipocyte differentiation [52]. These results suggest that the inhibitory effect of 4PF (**7**) on adipocyte differentiation may be associated with the suppression of MCE due to a decrease in *C/ebpδ* gene expression, which subsequently leads to the attenuation of lipid accumulation and the induction of PPARγ2 and C/EBPα. Other flavonoids also suppressed the process at the early stage of adipocyte differentiation. For example, fisetin and apigenin inhibited MCE as well as 4PF (**7**) [37,39]. Flavonoids with unique modification may exert their anti-obesity effects through the inhibition of MCE. Therefore, further studies are required to elucidate the molecular mechanism underlying the 4PF (**7**)-mediated suppression of MCE, which is essential for adipocyte differentiation.

Matsuda et al. showed the structural requirements of flavonoids for the adipogenesis in 3T3-L1 cells [53]. They found that most flavonoids with hydroxy groups lacked the effect, whereas flavonol with methoxy groups at the 3-position and the B-ring showed stronger effects. In contrast to flavonol, no significant difference was observed in the synthesized flavanone derivatives with methoxy groups in this study (Figure 3). Our results showed that 4PF (**7**) significantly inhibited lipid accumulation and adipocyte differentiation, whereas other flavanones had no or minimal effect on lipid accumulation among 15 flavanone derivatives (Figure 3). The substituent effects of the synthesized flavanones were independent of EDG or EWG, and the position of the substituent did not affect the inhibition of lipid accumulation and adipocyte differentiation; only 4PF (**7**) exerted a strong inhibitory effect. Although 4PF (**7**) with biphenyl structure is the only compound to have an inhibitory effect on lipid accumulation, there are no reports of biphenyl compounds on adipocyte differentiation and lipid accumulation. The natural flavanone naringenin has been reported to inhibit lipid accumulation and adipocyte differentiation by regulating AMPK activity [20]. Pinocembrin (**14**), used in this study, and naringenin are classified as (2S)-flavanones and their structural difference is the presence or absence of 4-hydroxyl groups in the B ring, respectively [54]. Although the inhibitory effect of naringenin on lipid accumulation and adipocyte differentiation was observed at 10 and 20 μM, the effect of pinocembrin (**14**) at 50 μM was statistically significant but minimal, suggesting that the introduction of 4-hydroxyl groups in the B ring might be involved in the inhibitory effect on adipocyte differentiation [20]. Saito et al. showed that flavanone has a PPARγ agonist activity at 30 μM by the mammalian two-hybrid system and enhanced adipocyte differentiation in 3T3-L1 cells [22], but the compound synthesized by our group did not exhibit the promotion of adipocyte differentiation at 50 μM (Figure 3). However, further verification is required to establish a structure–activity relationship.

In conclusion, the present study demonstrated that 4PF (**7**) inhibited lipid accumulation in 3T3-L1 cells by suppressing MCE and, subsequently, adipocyte differentiation. Consequently, the results from this study provide new insights into the biological activities of biphenyl structure and may serve as a novel therapeutic agent in the treatment of obesity and obesity-related diseases. Further studies are needed to investigate the relationship between the inhibition of adipocyte differentiation and the structure–activity relationship of flavanone derivatives.

## Figures and Tables

**Table 1:** Primers used in this study for qRT-PCR.

Gene	Primers	(5′–3′ Sequence)
*Ppary2*	Forward	GCTGTTATGGGTGAAACTCTG
	Reverse	ATAATAAGGTGGAGATGCAGG
*C/ebpα*	Forward	TGGACAAGAACAGCAACGAG
	Reverse	TCACTGGTCAACTCCAGCAC
*aP2*	Forward	ATGAAATCACCGCAGACGACAGGA
	Reverse	TGTGGTCGACTTTCCATCCCACTT
*Adiponectin*	Forward	AAGGACAAGGCCGTTCTCT
	Reverse	TATGGGTAGTTGCAGTCAGTTGG
*Glut4*	Forward	GCTTTGTGGCCTTCTTTGAG
	Reverse	CGGCAAATAGAAGGAAGACG
*C/ebpδ*	Forward	CCCCAAAGCTATGTGCCTTTC
	Reverse	CCTGGAGGGTTTGTGTTTTC
*C/ebpβ*	Forward	GGTTTCGGGACTTGATGCA
	Reverse	CAACAACCCCGCAGGAAC
*p16*	Forward	GTCGCAGGTTCTTGGTCACT
	Reverse	CGAATCTGCACCGTAGTTGA
*p21*	Forward	CGGTGGAACTTTGACTTCGT
	Reverse	CAGGGCAGAGGAAGTACTGG
*p27*	Forward	AGCAGTGTCCAGGGATGAGGAA
	Reverse	TTCTTGGGCGTCTGCTCCACAG
*p57*	Forward	AACGTCTGAGATGAGTTAGTTTAGAGG
	Reverse	AAGCCCAGAGTTCTTCCATCGT
*Pcna*	Forward	CCACATTGGAGATGCTGTTG
	Reverse	CAGTGGAGTGGCTTTTGTGA
*cMyc*	Forward	TCGCTGCTGTCCTCCGAGTCC
	Reverse	GGTTTGCCTCTTCTCCACAGAC
*CyclinA_2_*	Forward	TACCTGCCTTCACTCATTGCTGGA
	Reverse	ATTGACTGTTGGGCATGTTGTGGC
*CyclinD_1_*	Forward	TGCTGCAAATGGAACTGCTTCTGG
	Reverse	TACCATGGAGGGTGGGTTGGAAAT
*CyclinE*	Forward	AAGCCCTCTGACCATTGTGTCC
	Reverse	CTAAGCAGCCAACATCCAGGAC
*Cdk2*	Forward	CCCCAGAACCTGCTTATCAA
	Reverse	TGTGTTCCCCACACACTTA
*Cdk4*	Forward	TTTGTGGCCCTCAAGAGTGTGAGA
	Reverse	TCCTTAACAAGGCCACCTCACGAA
*Cdk6*	Forward	TAAAGCTGGCTGACTTTGGCCTTG
	Reverse	TCTGCAAAGATGCAACCGACACTC
*36b4*	Forward	AAGCGCGTCCTGGCATTGTCT
	Reverse	CCGCAGGGGCAGCAGTGGT

## Data Availability

The data supporting the conclusions of this work will be made available by the authors on request.

## Supplementary Materials

The following supporting information can be downloaded at: https://www.mdpi.com/article/10.3390/life14111446/s1, Figure S1: Effects of berberine on adipocyte differentiation; Figure S2: Effects of 4PF (**7**) and BBR on GAL4 PPARγ LBD-dependent luciferase activity. Figure S3: Effect of 4PF (**7**) on cell proliferation in HepG2 cells. HepG2 cells were treated with 4PF (**7**) (50 μM) or DMSO (Control) on day 0, 2, and 4.
