# Supplementary material for: Effects of Flavanone Derivatives on Adipocyte Differentiation and Lipid Accumulation in 3T3-L1 Cells
Source: Life (Basel). 2024 Nov 7;14(11):1446. doi: 10.3390/life14111446 (PMC11595554; doi:10.3390/life14111446)
Supplement: Supplementary file 1 [file life-14-01446-s001.zip › life-3277894-supplementary.pdf]

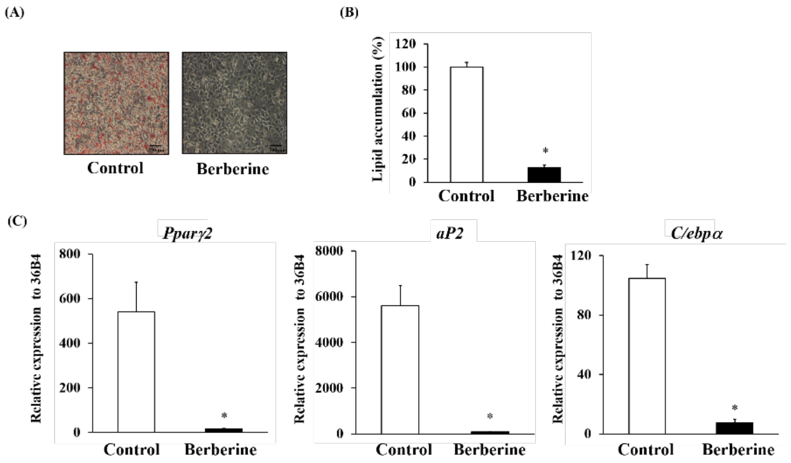


**Figure S1.** Effect of berberine on lipid accumulation in 3T3-L1 cells. 3T3-L1 cells were treated with berberine (4 μM) or DMSO (Control) during adipocyte differentiation. (**A**) Cells were stained with oil red O after 6 day of differentiation and were microscopically observed at 100 × magnification. (**B**) Intracellular lipids stained with oil red O dye were eluted, and their levels were quantified by measuring absorbance at 540 nm. The value of the Control cells was set to 100 (%). Data were represented as the means ± SD values of three independent experiments and were compared using an unpaired Student's *t*-test *P < 0.05. (**C**) mRNA expression levels of adipocyte differentiation-related genes. Total RNA was extracted and the expression of adipocyte differentiation-related genes was determined by qRT-PCR. The value of Control cells was normalized to 1. Data were compared using an unpaired Student's *t*-test *P < 0.05.

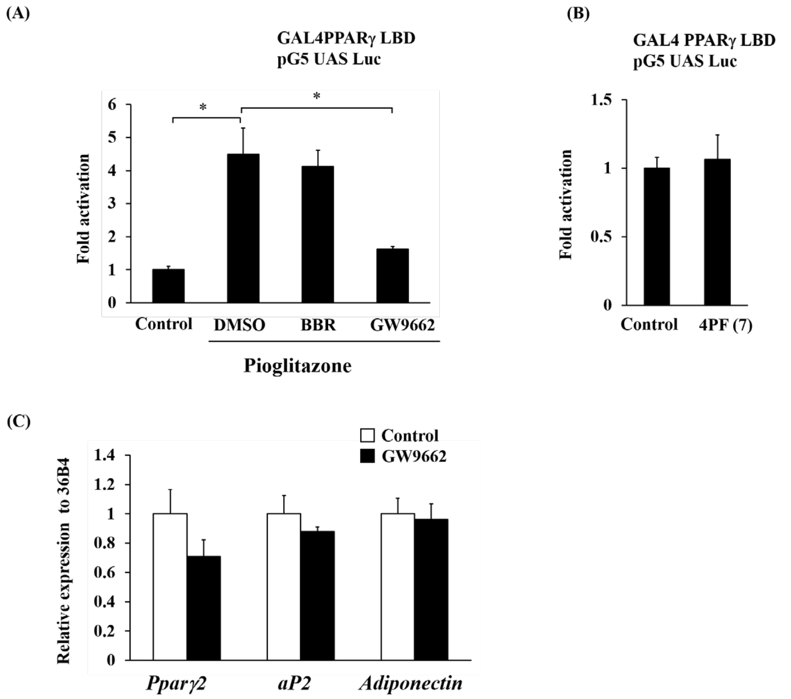


**Figure S2.** Effect of pioglitazone and 4PF (**7**) on GAL4 PPARγ LBD-dependent luciferase activity. Luciferase activity in 3T3-L1 preadipocytes transfected with the reporter plasmids containing pG5 UAS Luc, GAL4

PPARγ LBD. (**A**) Transfected cells were treated with BBR (4 μM), GW9662 (10 μM), and DMSO in the presence of pioglitazone (1 μM) for 18 h before luciferase assay. (**B**) Transfected cells were treated with 4PF (**7**) (50 μM) or DMSO for 18 h before luciferase assay. The value of Control (DMSO) cells was normalized to 1. Data were represented as the means ± SD values of three independent experiments and were compared using one-way ANOVA with Turkey's post-hoc test and *$P < 0.05$, relative to cells treated with both DMSO and pioglitazone (A) and using an unpaired Student's *t*-test *P < 0.05 (B). (**C**) Mature 3T3-L1 adipocytes were treated with GW9662 (10 μM) or DMSO (Control) for 24 h. Total RNA was extracted and the expression of adipocyte differentiation-related genes was determined by qRT-PCR. The value of Control cells was normalized to 1. Data were compared using an unpaired Student's *t*-test *P < 0.05.

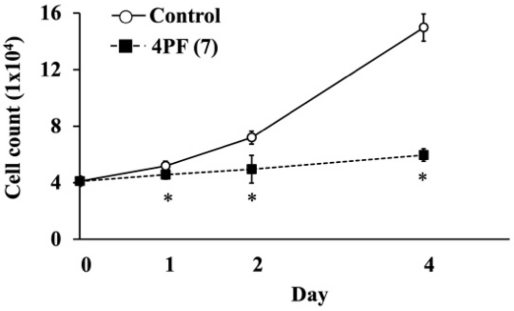


**Figure S3.** Effect of 4PF (**7**) on cell proliferation in HepG2 cells. HepG2 cells were treated with 4PF (**7**) (50 μM) or DMSO (Control) on day 0, 2, and 4. Cell counts were determined at the indicated time points. Data were compared using an unpaired Student's *t*-test *P < 0.05.
